# Cognitive function following breast cancer treatment and associations with concurrent symptoms

**DOI:** 10.1038/s41523-018-0076-4

**Published:** 2018-08-17

**Authors:** Kathleen Van Dyk, Julienne E. Bower, Catherine M. Crespi, Laura Petersen, Patricia A. Ganz

**Affiliations:** 1 UCLA Semel Institute for Neuroscience and Human Behavior, Los Angeles, CA USA; 2 UCLA Jonsson Comprehensive Cancer Center, Los Angeles, CA USA; 3 Departments of Psychology and Psychiatry/Biobehavioral Sciences, University of California-Los Angeles, Los Angeles, CA USA; 4 Department of Biostatistics, UCLA Fielding School of Public Health, Los Angeles, CA USA; 5 UCLA David Geffen School of Medicine, Los Angeles, CA USA; 6 UCLA Fielding School of Public Health, Los Angeles, CA USA

## Abstract

Cognitive changes after breast cancer treatment are often attributed to chemotherapy, without considering other important factors such as other treatments (e.g., surgery, radiation, endocrine therapy (ET)). We compared neuropsychological functioning in the domains of learning, memory, attention, visuospatial, executive function, and processing speed according to primary breast cancer treatment exposures in early survivorship, before the initiation of ET (*n* = 189). We were also interested in the association of neuropsychological functioning with select clinical, psychological, and behavioral factors. Compared to those who only underwent surgery (*n* = 28), all neuropsychological domain scores were comparable in a sample of breast cancer survivors with different treatment exposures, i.e., radiation therapy (*n* = 64), chemotherapy (*n* = 20), or both (*n* = 77), *p*’s < 0.05, adjusted for age, IQ, depression, and time since treatment completion. Physical fatigue, pain, and sleep correlated with several cognitive domains regardless of treatment exposure. There are minimal treatment-related neuropsychological differences on neuropsychological measures in early breast cancer survivorship, but the influence of other co-occurring symptoms warrants attention.

## Introduction

Cognitive dysfunction following breast cancer treatment is an important survivorship concern.^1^ Studies predominantly focus on chemotherapy treatment as the primary risk, although other treatments such as endocrine therapy (ET) and co-occurring factors likely also play a role.^2^ The mind body study (MBS) was a prospective, longitudinal, cohort study of early-stage breast cancer survivors (BCS) designed to assess the impact of ET on neurocognitive function; baseline analyses of this sample allows us to examine the effects of primary cancer treatments without the confound of concomitant ET. In prior baseline analyses, we found that higher subjective cognitive complaints were linked to combined chemotherapy and radiation therapy exposure.^3^ The current baseline study extends those findings by comparing neuropsychological functioning across treatment exposures; we further explored relationships with modifiable clinical, psychological, and behavioral factors.

## Results

Table 1 displays sample characteristics and cognitive outcomes by treatment exposure. We found comparable rates of impairment across treatment groups, and also failed to find any differences on neuropsychological domain scores between No Adjuvant and any adjuvant treatment group; effect sizes were small to negligible (see Supplementary Information for model details). Select clinical and psychosocial factors were correlated with several domains, notably the Pittsburgh Sleep Quality Index (PSQI), the Multidimensional Fatigue Symptom Inventory–Short Form (MFSI) Physical, and the Breast Cancer Prevention Trial Symptom Checklist (BCPT) Musculoskeletal Pain, see Table 2. Beck Depression Inventory, 2nd edition (BDI-II) did not correlate with any domain, and was included as an additional control. In additional exploratory analyses (data not presented) we examined linear regression models of domains that included treatment group and interactions between treatment group and each clinical/psychosocial factor, none of which emerged as significantly related to cognitive domains.

**Table 1:** Sample characteristics and cognitive performance by treatment group

			Treatment groups			
	Whole sample (*n* = 189)	(A) No Adjuvant (*n* = 28)	(B) Rad Only (*n* = 64)	(C) Chemo Only (*n* = 20)	(D) Chemo + Rad (*n* = 77)	*p* across groups
Age mean (SD)	51.35 (8.34)	51.57 (6.08)	53.88 (7.95)	46.95 (8.06)	50.31 (8.88)	0.001
Education, *n* (%)						
Less than college	34 (18%)	2 (7%)	14 (22%)	3 (15%)	15 (19%)	0.55
College degree	56 (30%)	8 (29%)	16 (25%)	8 (40%)	24 (31%)	
More than college	99 (52%)	18 (64%)	34 (53%)	9 (45%)	38 (49%)	
Marital status, *n* (%) married	124 (66%)	11 (39%)	24 (38%)	6 (30%)	24 (31%)	0.78
Race, *n* (%) White	151 (80%)	23 (82%)	53 (83%)	16 (80%)	59 (77%)	0.82
Annual income, *n* (%; *n* = 186)						
>$100,000	112 (60%)	18 (64%)	40 (65%)	11 (55%)	43 (57%)	0.72
<$100,000	74 (40%)	10 (36%)	22 (35%)	9 (45%)	33 (43%)	
Employment status, *n* (%) employed FT or PT	122 (66%)	20 (71%)	44 (69%)	10 (50%)	48 (63%)	0.38
Post-menopausal, *n* (%)	100 (53%)	15 (54%)	40 (62%)	5 (25%)	40 (52%)	0.03
Surgery						
Lumpectomy	125	4	63	0	58	< 0.01
Mastectomy	64	24	1	20	19	
Months since treatment completion, mean (SD)	1.197 (1.038)	2.48 (0.731)	0.960 (0.951)	1.282 (0.92)	0.908 (0.879)	<0.01
Anthracycline treatment, *n* (%)	24 (25%)	NA	NA	3 (15%)	21 (27%)	0.385
Stage at diagnosis, *n* (% of group)						
0	25 (13%)	14 (50%)	11 (17%)	0 (0%)	0 (0%)	<0.01
1	87 (46%)	13 (46%)	44 (69%)	7 (35%)	23 (30%)	
2	59 (31%)	1 (4%)	9 (14%)	12 (60%)	37 (48%)	
3	18 (10%)	0 (0%)	0 (0%)	1 (5%)	17 (22%)	
Endocrine therapy planned, *n* (%; *n* = 181)	129 (71%)	13 (48%)	50 (81%)	15 (75%)	51 (71%)	0.03
PSQI, mean (SD) (*n* = 186)	8.28 (3.46)	7.61 (3.79)	6.59 (3.36)	8.35 (4.03)	8.26 (3.31)	0.034
BDI-II, mean (SD)	8.85 (6.87)	7.36 (6.92)	6.86 (6.76)	12.75 (8.48)	10.04 (5.85)	<0.01
State anxiety inventory, mean (SD)	35.51 (8.75)	35.14 (8.24)	35.11 (9.38)	37.42 (9.95)	35.48 (8.16)	0.77
IQ WTAR, mean (SD) (*n* = 188)	114.28 (9.09)	116.61 (8.18)	114.37 (8.77)	111.10 (9.33)	114.18 (9.50)	0.23
MFSI total, mean (SD)	11.46 (19.34)	6.43 (19.02)	6.71 (19.12)	18.20 (20.16)	15.48 (18.32)	<0.01
MFSI mental, mean (SD)	5.51 (4.66)	3.61 (3.45)	4.11 (4.10)	7.30 (5.09)	6.91 (4.81)	<0.01
MFSI physical, mean (SD)	4.15 (4.29)	4.43 (4.83)	2.75 (3.54)	6.35 (3.50)	4.64 (4.52)	<0.01
BCPT scale, musculoskeletal pain mean (SD)	1.26 (0.95)	1.25 (0.88)	0.96 (0.75)	1.32 (0.75)	1.50 (1.10)	<0.01
# of impaired neuropsychological measures (*z* < −1.5)	1.40 (1.73)	1.46 (1.71)	1.17 (1.60)	1.15 (1.59)	1.64 (1.86)	0.39
# of impaired neuropsychological measures (*z* < −2)	0.075 (1.31)	0.64 (1.10)	0.66 (1.18)	0.65 (1.04)	0.88 (1.55)	0.71
Impaired by ICCTF guidelines *n* (% group)	89 (47%)	13 (46%)	27 (42%)	9 (45%)	40 (52%)	0.71
Neuropsychological domains						Standardized coefficients (95% CI) for A vs. B, A vs. C, and A vs. D^a^
Learning, mean (SD)^b^	0.39 (0.70)	0.47 (0.80)	0.43 (0.75)	0.38 (0.64)	0.32 (0.65)	0.03, 0.04, −0.02
Memory, mean (SD)^b^	0.21 (0.62)	0.19 (0.68)	0.29 (0.63)	0.16 (0.65)	0.17 (0.58)	0.07, 0.06, 0.14
Attention, mean (SD)^b^	0.46 (0.65)	0.66 (0.61)	0.49 (0.62)	0.31 (0.42)	0.40 (0.73)	−0.03, −0.08, −0.10
Visuospatial, mean (SD)^b^	−0.35 (0.74)	−0.24 (0.69)	−0.28 (0.77)	−0.54 (0.79)	−0.40 (0.72)	−0.06, −0.08, −0.11
Executive function, mean (SD)^b^	0.23 (0.76)	0.42 (0.86)	0.28 (0.73)	0.06 (0.64)	0.16 (0.76)	−.07, −0.09, −0.15
Processing speed, mean (SD)^b^	−0.06 (0.67)	0.08 (0.55)	−0.01 (0.71)	−0.12 (0.65)	−0.15 (0.68)	0.11, −0.05, 0.02

*BDI-II* Beck Depression Inventory, 2nd edition, *MFSI* Multidimensional Fatigue Symptom Inventory, *BCPT* Breast Cancer Prevention Trial Symptom Checklist, *PSQI* Pittsburgh Sleep Quality Index, *ICCTF* International Cognition and Cancer Task Force^13^

^a^Coefficients in linear models adjusted for age, IQ, BDI-II, and time since treatment completion; all *p*’s > 0.1

^b^Unadjusted scores

**Table 2:** Correlations between cognitive domains and other symptoms

	MFSI total	MFSI physical	MFSI mental	PSQI global	BCPT musculoskeletal pain
Learning					
Correlation	0.02	−0.08	−0.07	**−0.18**	**−0.15**
*p*	0.84	0.28	0.36	**0.02**	**0.04**
df	180	180	180	**177**	**180**
Memory					
Correlation	−0.01	−0.14	−0.04	**−0.22**	**−0.16**
*p*	0.92	0.06	0.59	**<0.01**	**0.04**
df	179	179	179	**177**	**179**
Attention					
Correlation	−0.13	**−0.25**	−0.09	**−0.29**	−0.13
*p*	0.09	**<0.01**	0.24	**<0.01**	0.08
df	179	**179**	179	**177**	179
Visuospatial					
Correlation	0.09	−0.08	0.04	−0.15	−0.12
*p*	0.24	0.30	0.64	0.05	0.11
df	179	179	179	177	179
Executive function					
Correlation	**−0.15**	**−0.25**	−0.12	**−0.17**	**−0.21**
*p*	**0.04**	**<0.01**	0.10	**0.02**	**<0.01**
df	**181**	**181**	181	**178**	**181**
Processing speed					
Correlation	−0.10	**−0.20**	−0.08	−0.14	−0.08
*p*	0.16	**<0.01**	0.30	0.06	0.29
df	181	**181**	181	178	181

Controls: Age, IQ, Time since TX, BDI-II

*BDI-II* Beck Depression Inventory, 2nd edition, *MFSI* Multidimensional Fatigue Symptom Inventory, *BCPT* Breast Cancer Prevention Trial Symptom Checklist, *PSQI* Pittsburgh Sleep Quality Index. Bold values indicate p< .05

## Discussion

Neuropsychological performance did not significantly vary based on primary breast cancer treatment exposure in this early survivorship period. Strengths of our study are assessment prior to ET exposure and the surgery-only comparison group. The current null findings are in contrast with our prior report of subjective cognition.^3^ Such inconsistency is not uncommon in survivorship studies, which compellingly portray the cognitive effects of cancer and its treatment by self-report, raising the possibility that neuropsychological methods may not be the most sensitive to these subtle effects.^4^

Neurocognitive function did correlate with physical fatigue, sleep quality, and pain, regardless of treatment. Fatigue is a known correlate of self-reported cognition in BCS, but pain and sleep disturbance are surprisingly understudied risks despite their prevalence in survivorship and known risk in other populations.^5–7^ Coefficients are small but portray a consistent pattern. Cognitive function is complex and multi-determined; it is important to exhaust all risks and opportunities for improvement, reflected in existing recommendations for multi-modal approaches to intervention.^8^

Study limitations include the predominantly white and highly educated sample aged 65 or younger. Additional work should examine the roles of socioeconomic factors, education, age, and comorbidity. The smaller sizes of the Chemo Only and No Adjuvant groups likely reduced power and we did not control for multiple comparisons, but effect sizes were nonetheless mostly negligible. Importantly, we did not have pre-treatment assessments, which would permit more precise inferences about treatment-related differences.

To conclude, we failed to find differences on neuropsychological test performance based on primary breast cancer treatment. The commonly reported symptoms of physical fatigue, pain, and sleep disturbance are promising targets for supporting cognitive health in BCS. Our future work will extend this baseline report to characterize the cognitive effects of ET and other risks over time.

## Methods

As previously described, three recruitment took place from 2007–2011 through clinical oncology practices and rapid case ascertainment using the Los Angeles County Surveillance, Epidemiology, and End Results Program registry with collaborating physicians and hospitals. This is a report of baseline data only; participants were age 21–65 years, had a recent early-stage breast cancer diagnosis, had completed primary treatment within the last 3 months but did not yet start ET. We excluded women with active psychotic or major depressive disorders, or any history of treatments or conditions with known effects on cognition or inflammation. The UCLA institutional review board approved the study and all participants provided written informed consent.

We obtained demographic and clinical information from medical records and self-report questionnaires. The following measures were used: BCPT,^9^ PSQI,^10^ MFSI,^11^ and BDI-II.^12^ We administered a neuropsychological battery composed of standardized clinical neuropsychological tests (see Supplementary Information); *z*-scores based on published normative data were averaged into domain scores.

All participants received surgery; those with no adjuvant treatment (No Adjuvant) were considered the no-treatment comparison group, and the rest were grouped by specific adjuvant therapy—those who received only chemotherapy (Chemo Only), only radiation therapy (Rad Only), or both chemotherapy and radiation (Chemo + Rad). We compared demographic, clinical, and impairment variables^13^ using two-sided analysis of variance (ANOVA) and chi-square tests. Multivariable linear regression models of neuropsychological domain scores controlled for age, intelligence quotient (IQ), time since treatment completion, and BDI-II, with treatment group dummy coded making No Adjuvant the reference group. We obtained partial correlations between cognitive domain scores and clinical and behavioral measures controlling for age, IQ, time since treatment completion and BDI-II. We used SPSS software (IBM SPSS Statistics for Windows, V.24.0. Armonk, NY: IBM Corp) and set statistical significance at *p* < 0.05.

### Data availability

On reasonable request, the data analyzed in this study are available from the corresponding author in accordance with institutional policies.

## Electronic supplementary material

Supplementary Information

## Electronic supplementary material

**Supplementary information** accompanies the paper on the *npj Breast Cancer* website (10.1038/s41523-018-0076-4).
