# Supplementary material for: Cognitive function following breast cancer treatment and associations with concurrent symptoms
Source: NPJ Breast Cancer. 2018 Aug 17;4:25. doi: 10.1038/s41523-018-0076-4 (PMC6098124; doi:10.1038/s41523-018-0076-4)
Supplement: Supplementary file 1 — Supplementary Information [file 41523_2018_76_MOESM1_ESM.pdf]

Supplemental Information

Neuropsychological Tests by Domain

Domain	Test/Measure
**Learning**	CVLT-II List A Total Trials 1-5
	WMS-III LM I
	BVMT-R Total Trials 1-3
**Memory**	CVLT-II List A Long Delay Free Recall
	WMS-III LM II
	BVMT-R Delayed Recall
	ROCFT 3-minute Delayed Recall
**Attention**	WAIS-III Digit Span, Coding, Letter-Number Sequencing
	TMT A completion time
	PASAT Trial 2 total errors
**Visuospatial**	ROCFT copy
	WAIS-III Block Design
**Executive Functioning**	TMT B
	Stroop Color Word Interference[1]
	Verbal Fluency (FAS)
**Processing Speed**	Grooved Pegboard[2]
	Stroop Word Reading & Color Naming

BVMT-R=Brief Visuospatial Memory Test-Revised[3]; CVLT-II=California Verbal Learning Test-II[4];TMT=Trail Making Test[5]; ROCFT=Rey-Osterreith Complex Figure Test[6,7]; Paced Auditory Serial Attention Test[8]; WAIS-III=Wechsler Adult Intelligence Scale-3rd Edition; WMS-III=Wechsler Memory Scale-3rd Edition[9]

1. Golden CJ, Freshwater SM. Stroop color and word test. 1978.
2. Klove H. Clinical Neuropsychology. *Med Clin North Am.* 1963;47:1647-1658.
3. Benedict RH. Brief Visuospatial Memory Test - Revised. Odessa, FL: Psychological Assessment Resources, Inc.; 1997.
4. Delis DC, Kaplan, E., Kramer, J.H. California Verbal Learning Test-II. 2nd ed. San Antonio, TX: The Psych. Corp.; 2000.
5. Reitan RM. Validity of the Trail Making Test as an indicator of organic brain damage. *Perceptual and Motor Skills.* 1958;8:271-276.
6. Strauss E, Sherman EMS, Spreen O. *A Compendium of Neuropsychological Tests.* 3rd ed. New York: Oxford University Press; 2006.
7. Mitrushina M, Boone KB, Razani J, D'Elia LF. *Handbook of normative data for neuropsychological assessment.* Oxford University Press; 2005.
8. Gronwell DMA. Paced auditory serial-addition task: A measure of recovery from concussion. *Perceptual and Motor Skills.* 1977;44:367-373.
9. Wechsler D. Wechsler Memory Scale. 3rd ed. New York: Psychological Corporation; 1997.

Full Statistics for Linear Models

Learning

Model ANOVA

Model		Sum of Squares	df	Mean Square	F	Sig.
1	Regression	10.675	7	1.525	3.452	0.002
	Residual	78.641	178	0.442		
	Total	89.316	185			

Coefficients

Model		Unstandardized Coefficients		Standardized Coefficients	t	Sig.	95.0% Confidence Interval for B	
		B	Std. Error	Beta			Lower Bound	Upper Bound
1	(Constant)	-2.427	0.736		-3.297	0.001	-3.880	-0.974
	Age	-0.004	0.006	-0.043	-0.581	0.562	-0.016	0.009
	BDI-II	-0.002	0.008	-0.016	-0.218	0.828	-0.017	0.013
	Time Since Tx	0.036	0.064	0.048	0.566	0.572	-0.090	0.162
	IQ	0.026	0.005	0.340	4.759	0.000	0.015	0.037
	Radiation Only v No Adjuvant	0.048	0.177	0.032	0.271	0.787	-0.302	0.398
	Chemo Only v No Adjuvant	0.086	0.216	0.038	0.397	0.692	-0.341	0.512
	Chemo and Radiation v No Adjuvant	-0.031	0.174	-0.022	-0.176	0.860	-0.375	0.313

Memory

Model ANOVA

Model		Sum of Squares	df	Mean Square	F	Sig.
1	Regression	5.574	7	0.796	2.220	.035
	Residual	63.486	177	0.359		
	Total	69.060	184			

Coefficients

Model		Unstandardized Coefficients		Standardized Coefficients	t	Sig.	95.0% Confidence Interval for B	
		B	Std. Error	Beta			Lower Bound	Upper Bound
1	(Constant)	-1.998	0.665		-3.007	0.003	-3.310	-0.687
	Age	-0.001	0.006	-0.016	-0.206	0.837	-0.012	0.010
	BDI-II	0.001	0.007	0.013	0.175	0.861	-0.012	0.015
	Time Since Tx	0.043	0.057	0.065	0.747	0.456	-0.070	0.156
	IQ	0.018	0.005	0.275	3.758	0.000	0.009	0.028
	Radiation Only v No Adjuvant	0.189	0.160	0.145	1.181	0.239	-0.126	0.504
	Chemo Only v No Adjuvant	0.113	0.195	0.057	0.579	0.563	-0.272	0.497
	Chemo and Radiation v No Adjuvant	0.090	0.157	0.072	0.569	0.570	-0.221	0.400

Attention

Model ANOVA

Model		Sum of Squares	df	Mean Square	F	Sig.
1	Regression	17.988	7	2.570	7.634	<0
	Residual	59.579	177	0.337		
	Total	77.567	184			

Coefficients

Model		Unstandardized Coefficients		Standardized Coefficients	t	Sig.	95.0% Confidence Interval for B	
		B	Std. Error	Beta			Lower Bound	Upper Bound
1	(Constant)	-2.063	0.644		-3.203	0.002	-3.333	-0.792
	Age	-0.015	0.005	-0.194	-2.805	0.006	-0.026	-0.004
	BDI-II	-0.008	0.007	-0.086	-1.235	0.218	-0.021	0.005
	Time Since Tx	0.034	0.056	0.048	0.605	0.546	-0.076	0.143
	IQ	0.030	0.005	0.422	6.297	0.000	0.021	0.039
	Radiation Only v No Adjuvant	-0.044	0.155	-0.032	-0.283	0.778	-0.349	0.262
	Chemo Only v No Adjuvant	-0.168	0.189	-0.080	-0.889	0.375	-0.540	0.205
	Chemo and Radiation v No Adjuvant	-0.138	0.152	-0.105	-0.906	0.366	-0.439	0.163

Visuospatial

Model ANOVA

Model		Sum of Squares	df	Mean Square	F	Sig.
1	Regression	11.779	7	1.683	3.361	0.002
	Residual	88.616	177	0.501		
	Total	100.395	184			

Coefficients

Model		Unstandardized Coefficients		Standardized Coefficients	t	Sig.	95.0% Confidence Interval for B	
		B	Std. Error	Beta			Lower Bound	Upper Bound
1	(Constant)	-3.160	0.785		-4.024	0.000	-4.709	-1.610
	Age	0.004	0.007	0.046	0.616	0.539	-0.009	0.017
	BDI-II	-0.004	0.008	-0.033	-0.442	0.659	-0.020	0.012
	Time Since Tx	-0.064	0.068	-0.081	-0.950	0.343	-0.198	0.069
	IQ	0.024	0.006	0.302	4.206	0.000	0.013	0.036
	Radiation Only v No Adjuvant	-0.092	0.189	-0.059	-0.488	0.626	-0.464	0.280
	Chemo Only v No Adjuvant	-0.193	0.230	-0.082	-0.840	0.402	-0.648	0.261
	Chemo and Radiation v No Adjuvant	-0.170	0.186	-0.113	-0.914	0.362	-0.537	0.197

Executive Function

Model ANOVA

Model		Sum of Squares	df	Mean Square	F	Sig.
1	Regression	26.467	7	3.781	8.410	<.000
	Residual	80.471	179	0.450		
	Total	106.938	186			

Coefficients

Model		Unstandardized Coefficients		Standardized Coefficients	t	Sig.
		B	Std. Error	Beta		
1	(Constant)	-3.503	0.743		-4.717	0.000
	Age	-0.009	0.006	-0.099	-1.449	0.149
	BDI-II	-0.007	0.008	-0.064	-0.934	0.351
	Time Since Tx	-0.057	0.064	-0.069	-0.893	0.373
	IQ	0.039	0.005	0.467	7.085	0.000
	Radiation Only v No Adjuvant	-0.117	0.177	-0.073	-0.662	0.509
	Chemo Only v No Adjuvant	-0.216	0.218	-0.088	-0.991	0.323
	Chemo and Radiation v No Adjuvant	-0.235	0.175	-0.153	-1.338	0.183

Processing Speed

Model ANOVA

Model		Sum of Squares	df	Mean Square	F	Sig.
1	Regression	1.839	7	0.263	0.960	0.462
	Residual	48.982	179	0.274		
	Total	50.821	186			

Coefficients

Model		Unstandardized Coefficients		Standardized Coefficients	t	Sig.	95.0% Confidence Interval for B	
		B	Std. Error	Beta			Lower Bound	Upper Bound
1	(Constant)	-0.241	0.579		-0.416	0.678	-1.384	0.902
	Age	-0.008	0.005	-0.123	-1.592	0.113	-0.017	0.002
	BDI-II	-0.007	0.006	-0.085	-1.099	0.273	-0.018	0.005
	Time Since Tx	0.037	0.050	0.066	0.749	0.455	-0.061	0.135
	IQ	0.004	0.004	0.078	1.044	0.298	-0.004	0.013
	Radiation Only v No Adjuvant	0.133	0.138	0.120	0.965	0.336	-0.139	0.406
	Chemo Only v No Adjuvant	-0.005	0.170	-0.003	-0.027	0.978	-0.340	0.331
	Chemo and Radiation v No Adjuvant	0.044	0.137	0.041	0.320	0.750	-0.226	0.314
